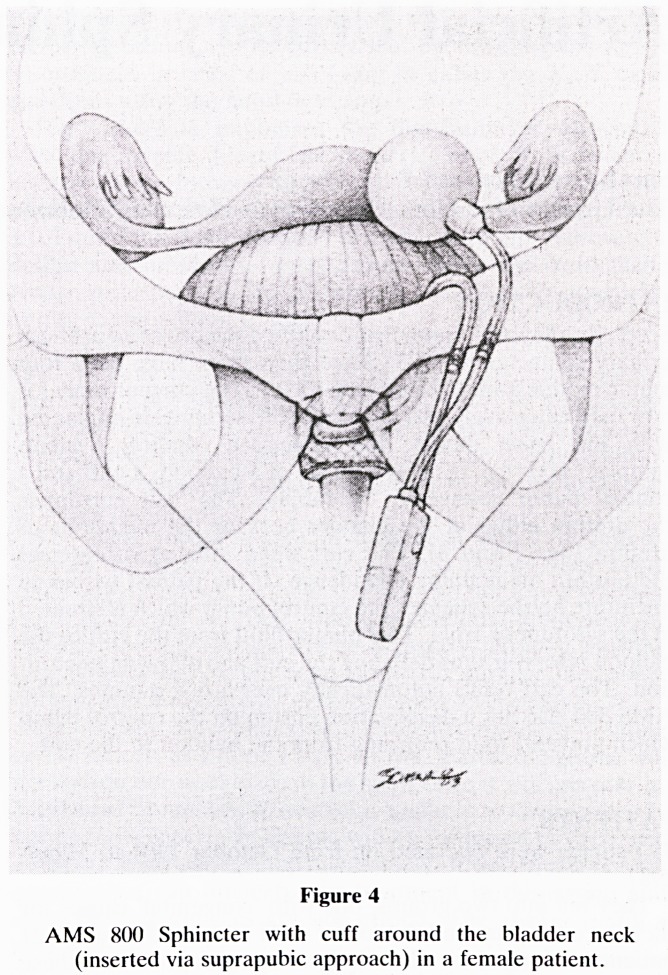# Artificial Urinary Sphincters—Early Experience

**Published:** 1989-05

**Authors:** Sarah Creighton, Paul Abrams

**Affiliations:** The Urodynamic Unit, Ham Green Hospital, Southmead Health Authority, Bristol; The Urodynamic Unit, Ham Green Hospital, Southmead Health Authority, Bristol


### Artificial Urinary Sphincters—Early Experience

**Sarah Creighton and Paul Abrams**
The Urodynamic Unit, Ham Green Hospital, Southmead Health Authority, Bristol

### INTRODUCTION

Scott, Bradley and Timm first described the use of an artificial urinary sphincter in 1973. Since then there have been four major modifications (Montague 1981). The current model is now used extensively. All patients in this series were implanted with the latest model, the American Medical Systems sphincter 800 (fig. 1). This consists of a balloon, a cuff and a control pump connected by tubing. The cuff surrounds the urethra either at the bladder neck or the membranous urethra (figs 2 and 3). The cuff when inflated compresses the urethra maintaining continence. If the patient wishes to micturate he/she squeezes the control pump which is situated in the scrotum or labia. This pumps fluid from the cuff to the balloon reservoir thus deflating the cuff and allowing micturition. The cuff refills automatically over a few minutes. The AMS 800 also has a deactivation button on the control pump which prevents fluid returning from the balloon to the cuff.

### PATIENTS

31 patients were operated on from October 1984 to March 1987.

They fell into two groups, one with 'congenital' causes for their incontinence and the other with 'acquired' causes. 21 patients belonged to the 'congenital' group. 17 of these patients had spina bifida of differing degrees of severity. 2 patients had sacral lipomata and 2 had sacral agenesis. 10 patients belonged to the 'acquired' group. 6 of these were incontinent following prostatectomy (4 transurethral and 2 retropubic prostatectomy). 1 was incontinent following radiotherapy for prostatic carcinoma and 3 following trauma due to road traffic accidents. Of these, all but one was neurologically normal.

The patients from the 'congenital' groups spanned an age range from 11–32. 7 patients were female and 14 male. The patients from the 'acquired' groups were all male and spanned an age range from 38–83 years.

### SELECTION CRITERIA

Patients were chosen for artificial sphincter implantation after careful assessment. Patients were referred from both inside the health authority and from further away. All patients were seen for full assessment at the Urodynamic Unit at Ham Green Hospital. They underwent a full history and examination followed by urodynamic studies. These comprised filling and voiding video cystometry, and urethral pressure profiles.

The following criteria were used for selection:-

(a) *Motivation*

All patients appeared well motivated. The procedures and extent of the surgery was fully explained. In the 'congenital'

**Figure 1**

The AMS 800 Artificial Urinary Sphincter

**Figure 2**

AMS 800 Sphincter with cuff around the bulbous urethra in a male patient (cuff inserted via perineal approach).

**Figure 3**

AMS 800 Sphincter with cuff around the bladder neck (inserted via suprapubic approach) in a male patient.

**Figure 4**

AMS 800 Sphincter with cuff around the bladder neck (inserted via suprapubic approach) in a female patient.

groups in particular, most of the patients were otherwise fit and leading normal lives apart from the incontinence. Achieving an acceptable sexual function was also a major motivating factor in many cases.

(b) *Intelligence*
Patients had to be of adequate intelligence to understand use of the sphincter. Manual dexterity was also required in order to be able to manipulate the control pump.

(c) *Mobility*
Mobility appears to be a factor of paramount importance. All patients in the 'congenital' groups were apparently mobile either independantly or with crutches and calipers. However 2 of the patients who were allegedly mobile at home were not witnessed to walk during their hospital admissions. Both of these patients developed considerable problems, eventually necessitating removal of the sphincters.

(d) *Urodynamic studies*
The aim of urodynamics are two-fold:
(i) Assessment of the urethra.

An artificial sphincter is only indicated if there is demonstrable incontinence due to sphincter weakness. However, in many patients with neuropathic conditions the sphincter, as well as being incompetent, is also obstructive during attempted micturition leading to significant residual urines. This is either due to sphincter contraction during voiding (detrusor–sphincter–dyssynergia) or to failure of sphincter relaxation (static distal sphincter obstruction) the bladder neck also requires assessment, although in most patients with neuropathic bladders the bladder neck is open for most of all of the filling phase.

(ii) Assessment of the bladder

The bladder should be classified according to

– sensation; abnormal sensation, which usually means a reduced sensation, may necessitate voiding by the clock.

– bladder capacity: a functional capacity of at least 400 ml. is needed to avoid frequency of micturition.

– detrusor compliance: the bladder pressure should remain low throughout filling. Low compliance, that is the steady increase of detrusor pressure during bladder filling, is dangerous to upper tract function and requires treatment (see below).

– detrusor instability: no contractions occur during bladder filling in the 'normal' patient. Most patients with neuropathic bladders have vesico urethral dysfunction and detrusor contractions (detrusor instability) is usual. If these contractions are greater than 20 cm of water in height, they require treatment (see below).

Vesico ureteric reflex was noted in several cases. In the 'congenital' groups all but 2 patients had features of detrusor instability. 4 patients were commenced on anticholinergics, and follow up urodynamics showed improvement. All had demonstrable stress incontinence and abnormal urethral pressures. Capacity ranged from 30–625 ml. but only 3 patients had a capacity below 200 ml. 3 of this group had ileal conduits and 1 had had an undiversion two years previously. In the 'acquired group' 6 patients had stable bladders and 4 had some features of instability. All had stress incontinence and abnormally low urethral pressure profile (U.P.P.) Capacity ranged from 250 to 600 ml.

### PROCEDURES

### 1. Pre-sphincter implantation

In male patients with incomplete emptying an external urethrotomy and bladder neck incision was necessary using the Collins diathermy knife. At the 7 o'clock position, a full thickness neck incision was carried out down to the verumontanum. A 12 o'clock anterior urethrotomy was also peformed from the bladder neck through to the membranous urethra.

### 2. Sphincter implantation

Perineal sphincters were inserted aroung the bulbous urethra beneath the bulbo-cavernosus muscle. The perineal route was only chosen if fibrosis at the bladder neck prevented insertion at that site. This insertion was performed as a one-stage procedure. The cuff was then left deactivated for six weeks, before the patient was brought back for simple activation by pressing the button on the control assembly in the scrotum.

Bladder neck sphincters were inserted in two ways. In most male patients (13) the cuff was placed around the urethra, above the pelvic floor but below the prostate, after division of the pubo-prostatic and pubo-urethral ligaments. When this was not possible the cuff was placed at the bladder neck, leaving the vasa and seminal vesicals posterior to the cuff.

In female patients the cuff was placed around the bladder neck and proximal urethra. In order to minimise the risk of erosion, in all recent patients, the cuff has been 'sandwiched' between two layers of omentum, having previously mobilised the omentum from the transverse colon.

If bladder augmentation (see below) was not performed, then the balloon and pump were also implanted at the primary procedure. The patient was then sent home for six weeks prior to activation of the sphincter.

The AMS 800 sphincter can be de-activated when the pump is inserted, by pressure on the valve mechanism within the pump. Once all post-operative swelling has settled the patient can then be readmitted for sphincter activation, achieved by firm pressure on the pump, via the skin.

### 3. Bladder Augmentation

When bladder capacity, bladder compliance or detrusor instability were significant problems the bladder was augmented. A variety of techniques was used. Where possible the ileum was used having split the bladder from ureteric orifice to ureteric orifice. The smaller the capacity, the greater the length of ileum used. The ileum was open fully, along its antimesenteric border, prior to its being sewn into the opened bladder. When augmentation was performed, then the balloon and pump were inserted at a second operation six weeks after the insertion of cuff and bladder augmentation. It was felt that this delay would reduce the risk of sphincter infection. Furthermore in some female patients the cuff was sufficient to make the patient continent.

None of the 'acquired' group underwent bladder augmentation, but 10 of the 'congenital' group underwent either an ileo or caeco-cystoplasty. In the 3 patients with ileal conduits, these were incorporated into the bladder during augmentation.

### RESULTS

(a) *Perineal Sphincters*

Of the 10 patients 6 were completely dry, 1 was damp, but improved on anticholinergics, and 2 were damp needing 1–2 pads per day which was still a marked improvement on their pre-operative condition. 1 patient remained wet and will be considered with the complications.

(b) *Bladder neck sphincters*

Of the 21 bladder neck sphincters 12 were dry and happy with use of the sphincters. 5 remained wet and will be considered with the complications. 3 sphincters had to be removed and 1 is awaiting completion.

All the male patients (25) except for one void normally. The remaining patient and all but two of the 6 female patients that were implanted use intermittent self-catheterisation, to ensure complete bladder emptying.

### COMPLICATIONS

### Peri-operative Problems

The majority of the surgery was uncomplicated although in three patients the urethra (3) and in one patient the rectum was breached. In each case the defect was sutured and omentum brought down to the defect. Two of these patients suffered post-operative infection and had their sphincter removed. Our current practice is to surround the urethra with omentum and come back later to implant the sphincter.

### Early post-operative problems

Four patients developed sphincter infections necessitating removal (2 discussed above). Of the remaining two patients, one a girl, eroded her cuff into the vagina, it was removed and the urethra surrounded by omentum. At subsequent surgery the correct plane was easy to find and she is now dry with a functioning sphincter. The fourth patient developed recurrent pseudomonous urinary infections post-operatively and eventually his sphincter became infected.

In two patients there was control pump failure and the patients returned to theatre to have the pump replaced.

### Late post-operative problems

Three patients developed recurrent stress incontinence due to the tissue within the cuff undergoing partial atrophy. This is presumed to be due to the pressure of the cuff. Two cuffs have been replaced with smaller sizes and both patients are now dry.

Two patients noticed pain at the site of the balloon. This pain was felt when the sphincter was used. The pain was presumed to be due to a tight fibrous capsule. In one patient this was divided with relief of symptoms.

Three patients developed leakage shown to be due to detrusor instability: one is controlled by anticholinergics and a second patient has been successfully treated by ileocystoplasty.

### DISCUSSION

It can be seen from the above results that in most cases artificial urinary sphincters work well. They can radically change the patient's life, their whole outlook and self-image. However it is obvious that there are still several problems to be overcome to improve the success rate of the procedure.

Lowering of the risk of infection is paramount to the success of the sphincter. Of the 4 infected patients, all eventually required removal of the sphincter. It is very distressing to a young patient who has undergone major surgery, to feel it has been in vain. Measures such as pre-operative urine culture and perioperative antibiotics cover are routine. In addition skin must be healthy and in good condition.

It would seem logical that the risk of infection could be reduced by performing the enterocystoplasty, (if this were done) at a seperate time to inserting the sphincter. However, of the 4 infected patients, 2 had bladder augmentation and 2 did not, so the evidence is inconclusive.

Assessment with regard to the need for augmentation can also be difficult. Urodynamics showed instability to be the cause of postoperative incontinence in several patients. In this patients pre-operative urodynamics showed either a stable bladder or minimal instability with good capacities, ranging from 330 to 500 ml. Urodynamics after sphincter insertion showed moderate to marked instability and capacities reduced, ranging from 200–330 ml. Anticholinergic therapy was beneficial, but enterocystoplasty may be necessary to achieve continence.

Careful study of these patients is necessary to perfect the use of the artificial urinary sphincter and fully utilize its benefit. Results are improving, and the delight of those patients continent, often for the first time in their lives, is the encouragement to continue.

### ACKNOWLEDGEMENT

Our thanks go to Southmead Health Authority and the South West Regional Health Authority for the support they have provided in enabling this Sphincter implantation programme to be established.

### REFERENCES

SCOTT F. B. BRADLEY W.E. TIMM G. W. 1973. Treatment of Urinary Incontinence by Inflatable Prosthetic Sphincter. *Urology* **1**, 250–252.

MONTAGUE D. K. 1981. The Scott–Bradley–Timm Artificial Urinary Sphincters *J. Urology* **125**, 796–798.

## Figures and Tables

**Figure 1 f1:**
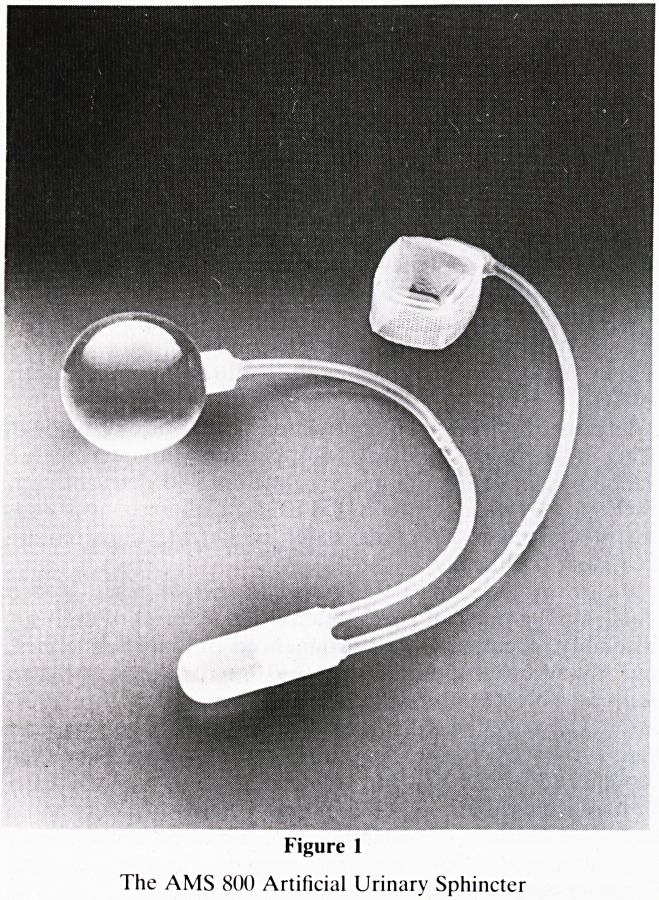


**Figure 2 f2:**
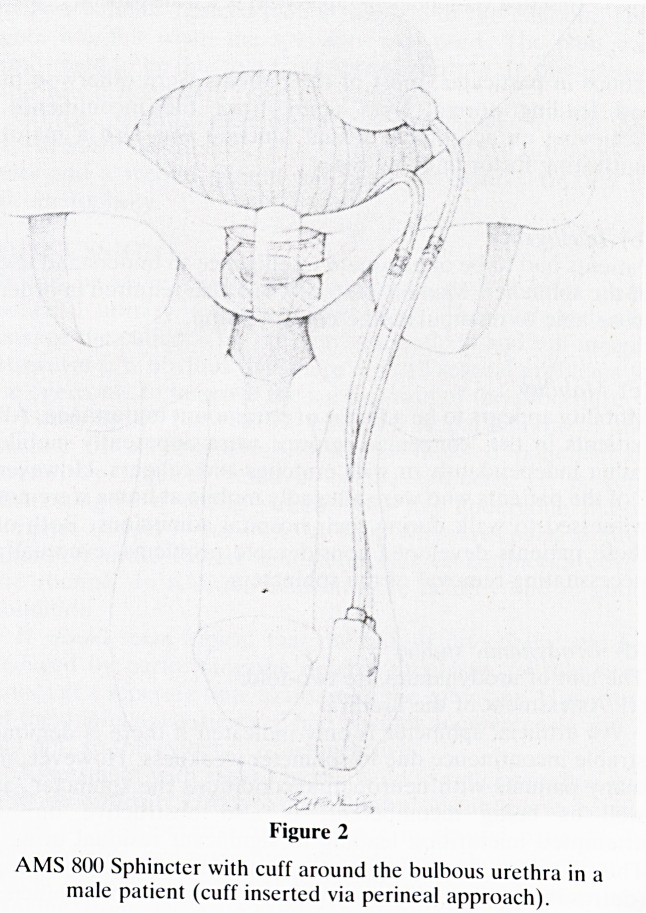


**Figure 3 f3:**
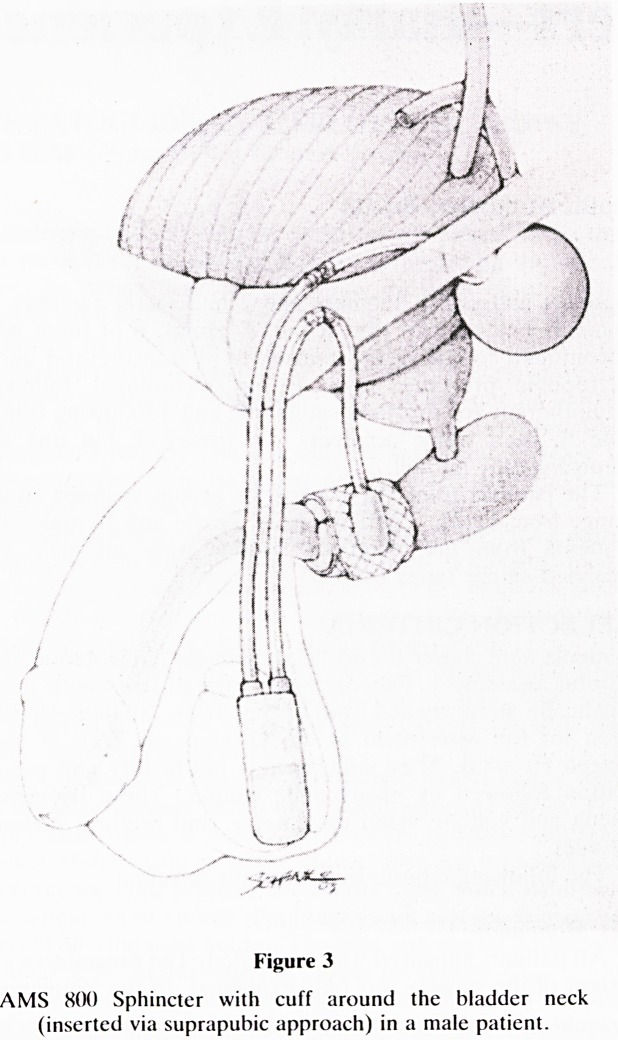


**Figure 4 f4:**